# COVID-19 pandemic-related depression and anxiety under lockdown: The chain mediating effect of self-efficacy and perceived stress

**DOI:** 10.3389/fpsyt.2023.1100242

**Published:** 2023-04-26

**Authors:** Luna Sun, Xiaoran Wang, Yi Hong, Chaoran Li, Wenfeng Zeng, Peng Liu, Yani Xiong, Yanping Chen, Yongjie Lian, Yunxia Wang

**Affiliations:** ^1^Department of Nautical Psychology, Faculty of Psychology, Naval Medical University, Shanghai, China; ^2^Department of Special Medical, Shanghai Fourth People’s Hospital, School of Medicine, Tongji University, Shanghai, China; ^3^Academic Affairs Office, Naval Medical University, Shanghai, China

**Keywords:** COVID-19, depression, anxiety, perceived stress, self-efficacy, perceived social support

## Abstract

**Objective:**

In early March 2022, the highly contagious Omicron variant rapidly emerged in Shanghai. This study aimed to explore the prevalence and associated factors of depression and anxiety in isolated or quarantined populations under lockdown.

**Methods:**

A cross-sectional study was conducted between May 12 and 25, 2022. The depressive and anxiety symptoms, perceived stress, self-efficacy and perceived social support in the 167 participants under isolated or quarantined were examined using the Patient Health Questionnaires-9 (PHQ-9), the Generalized Anxiety Disorder-7 (GAD-7), the Perceived Stress Scale-10 (PSS-10), the General Self-Efficacy Scale (GSES) and the Perceived Social Support Scale (PSSS). Data on demographic information were also collected.

**Findings:**

The prevalence of depression and anxiety in isolated or quarantined populations was estimated to be 12 and 10.8%, respectively. Higher education level, being healthcare workers, being infected, longer duration of segregation and higher perceived stress level were identified as risk factors for depression and anxiety. Furthermore, the relationship between perceived social support and depression (anxiety) was mediated not only by perceived stress but also the chain of self-efficacy and perceived stress.

**Conclusion:**

Being infected, higher education level, longer duration of segregation and higher perceived stress were associated with higher levels of depression and anxiety among isolated or quarantined populations under lockdown. The formulation of psychological strategies that promote one’s perceived social support and self-efficacy as well as reduce perceived stress is supposed to be drawn.

## Introduction

The outbreak of coronavirus disease 2019 (COVID-19) was announced to constitute a public health emergency of international concern by the World Health Organization (WHO) on 30 January, 2020 (1), and it has continued to rampage globally up to now. Shanghai, the largest financial and economic hub of China, was placed under a citywide lockdown due to its worst COVID-19 outbreak caused by the highly contagious Omicron variant in early March 2022 (2). As of May 31, 2022, a total of 626,000 COVID-19 infections, including confirmed locally transmitted cases and asymptomatic carriers, were reported in Shanghai (3). The global public health should be alarmed by the increased transmissibility and immune evasive properties of SARS-CoV-2 variants.

Effective prevention and control of COVID-19 has been a health issue of grave concern worldwide (4). Measures such as lockdowns, isolation, quarantine and social distancing have been implemented by many countries and regions in reaction to the COVID-19 pandemic (5–7). There is no denying that these measures are acknowledged as practical containment strategies for the pandemic, but the negative impact of these measures on individuals, societies, and the economy should be considered with caution (8–10). Although isolation and quarantine are conceptually distinct, both involve the separation from normal populations and the restriction of movement to prevent or eliminate the spread of infection or contamination (11). A growing number of studies have confirmed that people who underwent isolation or quarantine during the COVID-19 pandemic were subjected to a tremendous psychological and physical burden, which gave rise to a wide variety of dramatic and long-lasting psychological distress, such as depression, anxiety, posttraumatic stress disorder, insomnia and high perceived stress (12–16). It has been documented by a global-scale study that the COVID-19 pandemic resulted in a remarkable increase in the prevalence and burden of major depressive disorder (a 28% increase) and anxiety disorders (a 26% increase) during 2020 (17), both of which ranked among the leading causes of the global burden of disease even before the COVID-19 pandemic (18). Moreover, a national study on the mental health impact of COVID-19 pandemic post-lockdown demonstrated a high prevalence of depression (39%) and anxiety (42%) in the adult US population (19). A systematic review revealed that the prevalence of anxiety and depression during the initial COVID-19 lockdown in the United Kingdom was 31 and 32%, respectively, showing a substantial increase compared with the prevalence of pre-pandemic (20). Consequently, the development of timely and effective psychological interventions for individuals in isolation or quarantine is a critical component of the COVID-19 management. There are growing appeals for prioritizing mental health from the very start to identify and protect vulnerable populations and enhance long-term resilience against future crises (21).

However, due to the current prioritization of limited medical resources for the containment of COVID-19 and the treatment of infected patients, obtaining adequate resources for mental health services remains to be a formidable challenge. Targeting psychological support with limited resources for diverse populations impacted by the COVID-19 pandemic is, therefore, of essential significance. Lazarus has proposed that cognitive appraisal mediates the relationship between stressors and emotional experience, which accounts for emotional responses varying from person to person even under the same or similar conditions (22). Hence, developing psychological interventions that emphasize promoting individuals’ personal resources and altering their negative cognitive appraisals may contribute to emotion regulation under public health emergencies such as the COVID-19 pandemic. In view of the circumstance of lockdown that may trigger social and emotional isolation, social support and self-efficacy are vital external and internal resources, respectively, that many researches have demonstrated their association with depression and anxiety (23–25).

Social support is a multidimensional concept characterized by the emotional, instrumental, and informational support from families, friends and important others (26), which was identified as a protective factor against depression and anxiety during the COVID-19 pandemic (12, 24). Further, a review on the association between social support and depression provided evidence for the protection of social support for depression across all ages (27). Unlike received social support, which is described as objective and specific assistance from social networks, perceived social support highlights more the subject perception and evaluation of the available resources and supports from social relations (28), which relates more tightly to cognition. Therefore, the current study focused on perceived social support to investigate how it exerts an effect on depression and anxiety among isolated or quarantined populations during the COVID-19 pandemic. Self-efficacy was defined as the belief in one’s competence and efficiency to successfully tackle tasks by Bandura (29). Existing research have proved a negative correlation between self-efficacy and negative mental health outcomes resulted from COVID-19 pandemic, such as stress, depression, anxiety and fear (23, 30). It is suggested that higher self-efficacy can prevent poor psychological outcomes during COVID-19 pandemic (31). A full mediating effect of self-efficacy between social support and negative emotions (depression and anxiety) was also demonstrated in patients with prostate cancer (32). Furthermore, people under isolation or quarantine during the COVID-19 pandemic were confronted with stressors in diverse aspects, which tended to generate high level of perceived stress (33, 34). There was evidence that perceived stress worked as a mediator for longitudinal negative effects (containing depression and anxiety) of the COVID-19 lockdown (35). It has also been determined the potential role of perceived stress as a mediator between social support, self-efficacy (measured as coping self-efficacy) and depressive symptoms (36). In consequence, it is reasonable to suppose that improving one sense of perceived social support and self-efficacy, as well as reducing perceived stress will mitigate COVID-19-related psychological consequences of depression and anxiety under the COVID-19 pandemic.

Notwithstanding, no study to date, to our knowledge, has yielded the association among perceived stress, self-efficacy, perceived social support, depression and anxiety in isolated or quarantined population during the COVID-19 pandemic, specifically the mediating effects of self-efficacy and perceived stress. The primary objectives for this study were twofold: (1) to determine the prevalence and associated factors of depression and anxiety among the population isolated or quarantined under Shanghai lockdown; (2) to examine how perceived social support, self-efficacy and perceived stress affect the level of depression and anxiety, and to determine the potential mediating effects. Previous literature on COVID-19 showed that demographic factors (i.e., age, gender and educational level), presence of family or pets, being infected or not and duration of isolation or quarantine were significantly correlated to depression and anxiety (23, 37, 38). Therefore, these variables were hypothesized as potential associated factors in this study. Further, it was hypothesized that self-efficacy and perceived stress mediated the association between perceived social support and the level of depression (anxiety).

## Methods

### Participants and procedure

This was a cross-sectional study conducted at a centralized isolation and treatment site under lockdown in Shanghai between May 12 and 25, 2022. During the survey period, approximately 1,000 individuals were isolated or quarantined at the site, including COVID-19-positive patients and healthcare workers caring for the patients. The sample size was calculated with α set as 0.05, β as 0.2, and the overall prevalence of mood disorders (depression and anxiety) estimated as 35%, which came from a nationwide large-scale survey of psychological distress among Chinese during the COVID-19 epidemic (39). Thereby, a minimum sample size of 151 was required in this study. The following inclusion criteria were adopted for the recruitment of eligible participants: (1) being isolated or quarantined at the isolation site during the Shanghai lockdown; (2) aged ≥18 years old; (3) normal ability of speech, comprehension and expression; and (4) volunteering to participate in the study. Respondents who had previously been diagnosed with mental illness or in serious physical condition were excluded.

To minimize the risk of cross-infection, the questionnaire survey was conducted on an online platform^1^ *via* personal smartphone. Data collection fell primarily under the purview of a medical assistant with professional psychological training. All participants provided written or verbal informed consent prior to participation in the study after the medical assistant explained the nature of the study. It was an anonymous survey, but participants were asked to give their phone number voluntarily if they needed emotional or psychological support. Ethical permission for the study was granted by the Ethics Committee of Naval Medical University.

### Measures

Participants were asked to complete a series of questionnaires. Information on demographic characteristic were collected from every participant: age, gender, education level, marital status, employment status, smoking status, presence of family or pets, current status (patients or healthcare workers), infection status and duration of segregation (isolation or quarantine). Furthermore, the levels of depression, anxiety, perceived stress, self-efficacy and perceived social support were measured using corresponding validated scales.

### Depression

The Patient Health Questionnaire-9 (PHQ-9) (40) is a self-administered screening tool for depression, measuring to what extent an individual has been bothered by depressive symptoms during the past two weeks. The scale is consisted of 9 items, each on a Likert scale from “0” (not at all) to “3” (nearly every day), with an aggregate score ranging from 0 to 27. A higher score indicates higher level of depression and a cutoff score of 10 has been clinically validated for major depression with a sensitivity of 88% and a specificity of 88% ^[2]^. This scale has been well applied in the general Chinese population with great reliability and validity (41). In the current study, a PHQ-9 score of 10 or higher was indicative of having elevated depressive symptoms (probable depression) and the Cronbach’s α for internal consistency was 0.880.

### Anxiety

Also with a focus on the past two weeks, the Generalized Anxiety Disorder-7 (GAD-7) (42) is a 7-item self-administered scale assessing the frequency with which an individual has been bothered by anxiety symptoms, with each item on a Likert scale from 0 (not at all) to 3 (nearly every day). The total score of all items ranges from 0 to 21 and a higher score indicates higher level of anxiety. A cut point of 10 on the GAD-7 has been recommended for screening generalized anxiety disorders with a sensitivity of 89% and a specificity of 82% in a large-sample research (42). The Chinese version of this scale has been widely applied in clinical institutions and scientific researches (43). Thus, a GAD-7 score of 10 or higher was defined as having elevated anxiety symptoms (probable anxiety) in this study and the Cronbach’s α for internal consistency was 0.936.

### Perceived stress

Perceived stress was assessed by the Perceived Stress Scale-10 (PSS-10) (44), a self-report instrument measuring the level of perceived stress over the past month. It consists of 10 items on a 5-point Likert scale from 0 (never) to 4 (very often), with a total score of 0–40 and a higher score reflecting higher perception of stress. The PSS-10 has shown superior psychometric properties across a range of populations (45) and Chinese version of the scale has also obtained satisfactory psychometric properties (46). The Cronbach’s α for internal consistency in the current sample was 0.799.

### Self-efficacy

The self-efficacy was measured by the General Self-Efficacy Scale (GSES) (47), a self-report scale developed by Schwarzer and Jerusalem, which is composed of 10 items for assessing one’s generalized sense of self-efficacy regarding resourcefulness and processing power. A 4-point Likert scale is used for each item, from “1” (not at all true) to “4” (exactly true), with a total score ranging from 0 to 40 and a higher score reflecting higher level of self-efficacy. The Chinese version of the scale has demonstrated good reliability and validity (48). The Cronbach’s α for internal consistency in the current sample was 0.922.

### Perceived social support

The Chinese version of the Perceived Social Support Scale (PSSS) was translated and revised by Jiang Qianjin (49) based on the Multidimensional Scale of Perceived Social Support (MSPSS) developed by Zimet et al. (50), which evaluates an individual’s perception of support from family, friends and significant others from a subjective perspective. The scale consists of 12 items, each of which rated on a Likert scale from 1 (very strongly disagree) to 7 (very strongly agree), with an aggregate score ranging from 12 to 84 and higher scores indicating higher levels of perceived social support. Multiple samples have demonstrated the scale’s reliability and validity to be high (50, 51). The Cronbach’s α for internal consistency in the current sample was 0.933.

### Statistical analysis

Continuous variables were presented as mean and standard deviation (SD), and categorical variables were presented as frequency and percentage. Normality tests were performed before further analysis. Then, independent *t*-tests, one-way analysis of variance (ANOVA), or Mann–Whitney *U* tests were employed to compare the differences between subgroups on depression and anxiety levels (by the scores of PHQ-9 and GAD-7), as appropriate. To examine the association between psychological variables, the Pearson correlation coefficient for continuous variables was calculated. Multiple linear regression analysis (enter) was conducted to identify associated factors for depression and anxiety, with the scores of PHQ-9 and GAD-7 entered as the dependent variables and potential associated variables entered as independent variables. All statistical analyses were performed by SPSS 22.0 (IBM, Chicago, United States), and all tests were two-tailed with the significance level set at *p* < 0.05. In addition, given the small sample size of our study, bias-corrected (BC) bootstrap with 95% confidence interval (CI) based on 5,000 bootstrap samples using the Model 6 in PROCESS macro for SPSS was employed to examine the mediating effects (52). If a 95% BC bootstrap CI does not cover zero, the mediating effect is supported; otherwise, then it is not supported (53).

## Results

**Figure 1:**
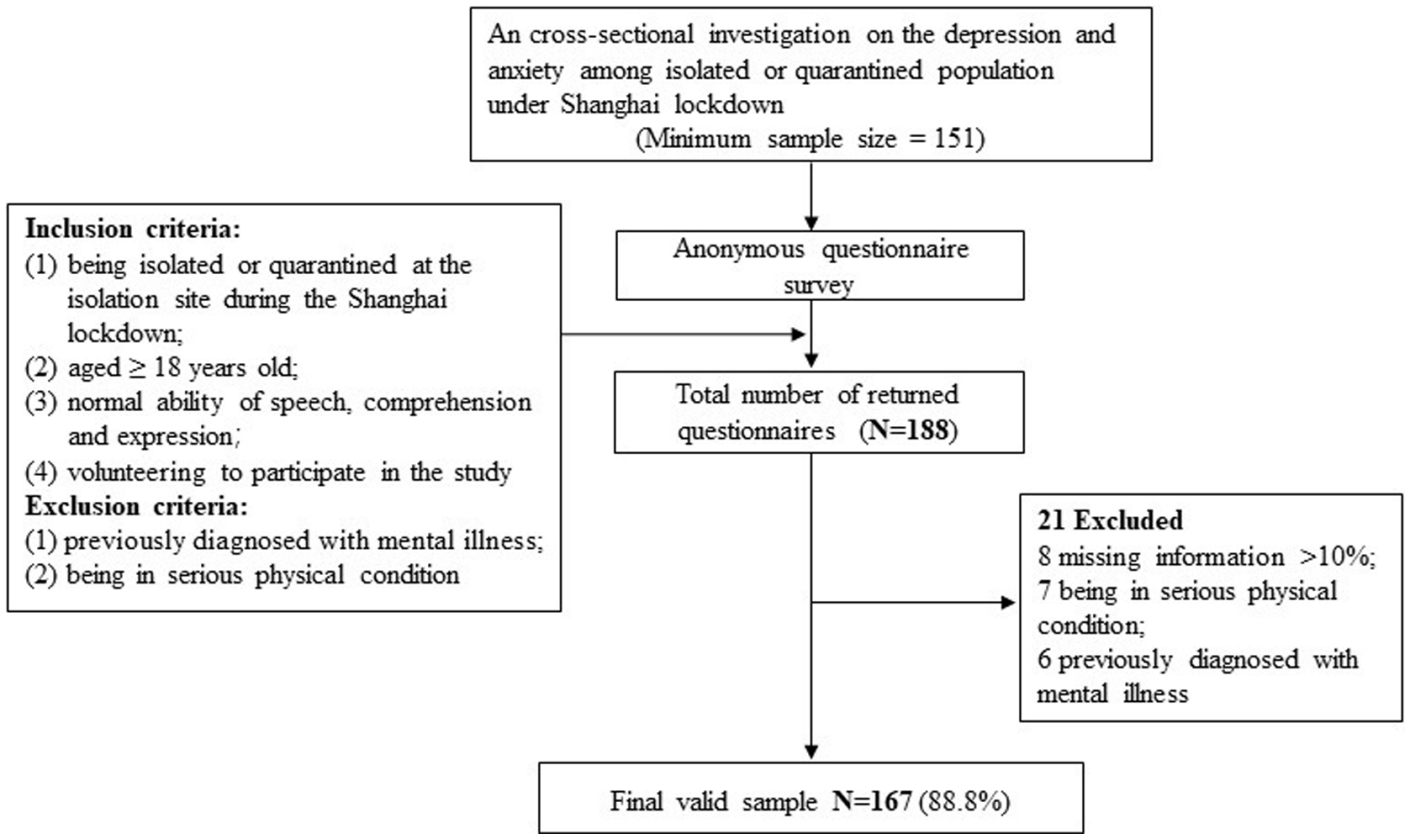
Flow chart of the enrollment of study participants.

The process of participant recruitment is illustrated by Figure 1. Initially, 188 respondents completed the questionnaire survey. In conjunction with preliminary questionnaire collation, 8 were excluded due to missing information exceeding 10%, 7 were excluded due to serious physical condition and 6 were excluded due to a previous diagnosis of mental illness. Finally, a total of 167 participants were enrolled in the analysis, with a valid response rate of 89.4%.

### Sample characteristics and the prevalence of depression and anxiety

Table 1 presents the demographic characteristics and the prevalence of probable depression and anxiety of the enrolled participants. The final sample was made up of 63 (37.7%) males and 104 (62.3%) females with an average age of 34.41 (SD, 11.9) years and the majority (79.6%) between the ages of 18 and 44. Among the participants, most (71.9%, 120 of 167) held a university or college education or higher, nearly a half (52.1%, 87 of 167) were married, 79.6% (133 of 167) were employed, and the vast majority (84.4%, 141 of 167) were nonsmokers. By the time the study was conducted, the mean duration of segregation for all participants was 21.35 (SD, 15.35) days, with more than a half (59.3%, 99 of 167) exceeding 14 days. During the segregation period, 44.3% (74 of 167) were accompanied by family or pets. Additionally, of the 167 participants, 73 (43.7%) were patients and 94 (56.3%) were healthcare workers. Totally, there were 76 (45.5%) participants being infected with COVID-19, among whom 3 were healthcare workers. With a cut point of 10, the prevalence of probable depression and anxiety determined to be 12.0 and 10.8%, respectively.

**Table 1:** Demographic characteristics and mood disorders of the participants (*N* = 167).

Variables	*n*	%
Age (M, SD)	34.41	11.90
18–44	133	79.6
45–59	25	15.0
60–74	9	5.4
Gender		
Male	63	37.7
Female	104	62.3
Education level		
High school or below	47	28.1
University or college	74	44.3
Postgraduate or above	46	27.5
Marital status		
Unmarried	80	47.9
Married	87	52.1
Employment status		
Employed	133	79.6
Unemployed	34	20.4
Smoking status		
Nonsmoker	141	84.4
Smoker	26	15.6
Presence of family or pets		
Yes	74	44.3
No	93	55.7
Current status		
Patients	73	43.7
Healthcare workers	94	56.3
Infected or not		
No	91	54.5
Yes	76	45.5
Duration of segregation (days, M, SD)	21.52	15.35
≤ 14	68	40.7
> 14	99	59.3
Depression (M, SD)	5.16	4.69
PHQ-9 ≥ 10	20	12.0
PHQ-9 < 10	147	88.0
Anxiety	4.14	4.43
GAD-7 ≥ 10	18	10.8
GAD-7 < 10	149	89.2

M, Mean. SD, Standard deviation. PHQ-9, Patient Health Questionnaire-9. GAD-7, Generalized Anxiety Disorder-7.

### Influence of demographic characteristics on depression and anxiety level

The differences in depression and anxiety levels between the subgroups stratified by demographic characteristics were tested (Supplementary Table S1). It was found that healthcare workers (*p* = 0.030), those who were infected (*p* = 0.033) and had been segregated for more than 14 days (*p* = 0.002) reported higher level of anxiety. However, there was no significant difference on depression level between the above subgroups. Moreover, no difference was observed on depression or anxiety levels between participants of different age distribution, gender, education level, marital status, employment status, smoking status, presence of family or pets and COVID-19 infection status.

### Correlations between depression, anxiety, perceived stress, self-efficacy, and perceived social support

The levels of depression, anxiety, perceived stress, self-efficacy and perceived social support were measured by PHQ-9, GAD-7, PSS-10, GSES, and PSSS, respectively. Table 2 displays the results of the bivariate correlation analysis of depression, anxiety, perceived stress, self-efficacy and perceived social support, along with their corresponding scale scores. There was a significantly positive correlation between the level of depression and anxiety (*r* = 0.790, *p* < 0.01), and perceived stress (*r* = 0.702, *p* < 0.01), as well as a significantly negative correlation between the level of depression and self-efficacy (*r* = −0.350, *p* < 0.01), and perceived social support (*r* = −0.269, *p* < 0.01). Similarly, there was a significantly positive correlation between the level of anxiety and perceived stress (*r* = 0.663, *p* < 0.01), and a significantly negative correlation between the level of anxiety and self-efficacy (*r* = −0.257, *p* < 0.01), and perceived social support (*r* = −0.207, *p* < 0.01). Furthermore, the level of perceived stress was negatively correlated with self-efficacy (*r* = −0.536, *p* < 0.01) and perceived social support (*r* = −0.365, *p* < 0.01). The level of self-efficacy was positively correlated with perceived social support (*r* = 0.344, *p* < 0.01).

**Table 2:** Correlations between depression, anxiety, perceived stress, self-efficacy and perceived social support.

	1	2	3	4	5
1. Depression	1				
2. Anxiety	0.790^**^	1			
3. Perceived stress	0.702^**^	0.663^**^	1		
4. Self-efficacy	−0.350^**^	−0.257^**^	−0.536^**^	1	
5. Perceived social support	−0.269^**^	−0.207^**^	−0.365^**^	0.344^**^	1
Mean	5.16	4.14	13.37	25.94	61.04
Standard deviation	4.69	4.43	6.29	6.34	13.18

^**^p < 0.01.

### Associated factors for depression and anxiety

Table 3 shows the results of the multiple linear regression models examining the associated factors for depression and anxiety level. It revealed that education level, being healthcare workers, infection status, and the level of perceived stress were significant factors for depression level. Participants with education level of university or college (*B* = 1.500, 95% CI [0.080, 2.919], *p* = 0.039) and postgraduate or above (*B* = 2.260, 95% CI [0.265, 4.256], *p* = 0.027), those being healthcare workers (*B* = 3.017, 95% CI [0.670, 5.364], *p* = 0.012), being infected with COVID-19 (*B* = 4.028, 95% CI [1.598, 6.458], *p* = 0.001) and possessing higher level of perceived stress (B = 0.547, 95% CI [0.448, 0.645], *p* < 0.001) tended to report higher level of depression. Broadly speaking, these variables contributed significantly to the amount of variance in depression level (*R*^2^ = 56.8%, Adjusted *R*^2^ = 52.8%, *F* = 14.290, *p* < 0.001). As for anxiety, the results indicated that higher level of anxiety was significantly associated with education level of university or college (*B* = 1.336, 95% CI [0.012, 2.659], *p* = 0.048) and postgraduate or above (*B* = 3.458, 95% CI [1.598, 5.317], *p* < 0.001), being healthcare workers (*B* = 2.275, 95% CI [0.087, 4.462], *p* = 0.042), being infected with COVID-19 (*B* = 3.561, 95% CI [1.295, 5.826], *p* = 0.002), longer duration of segregation (*B* = 0.056, 95% CI [0.012, 0.100], *p* = 0.012) and higher level of perceived stress (*B* = 0.540, 95% CI [0.448, 0.633], *p* < 0.001). Likewise, the amount of variance in anxiety level accounted for by these variables was statistically significant (*R*^2^ = 57.9%, Adjusted *R*^2^ = 54.0%, *F* = 14.906, *p* < 0.001).

**Table 3:** Multiple linear regression analysis of associated factors for depression and anxiety level.

	Depression level			Anxiety level		
	*B*	95% CI	*p* value	*B*	95% CI	*p* value
Age	0.012	[−0.051, 0.074]	0.717	0.039	[−0.020, 0.097]	0.191
Gender						
Male	Reference					
Female	−0.873	[−2.083, 0.337]	0.156	−0.006	[−1.134, 1.122]	0.992
Education level						
High school or below	Reference					
University or college	1.500	[0.080, 2.919]	0.039	1.336	[0.012, 2.659]	0.048
Postgraduate or above	2.260	[0.265, 4.256]	0.027	3.458	[1.598, 5.317]	<0.001
Marital status						
Unmarried	Reference					
Married	−1.131	[−2.655, 0.393]	0.145	−0.413	[−1.834, 1.007]	0.566
Employment status						
Employed	Reference					
Unemployed	−0.438	[−1.742, 0.865]	0.507	−0.145	[−1.360, 1.070]	0.814
Smoking status						
Nonsmoker	Reference					
Smoker	−0.431	[−1.955, 1.092]	0.577	−1.212	[−2.633, 0.208]	0.094
Presence of family or pets						
Yes	Reference					
No	−0.457	[−1.582, 0.669]	0.424	0.060	[−0.989, 1.110]	0.910
Current status						
Patients	Reference					
Healthcare workers	3.017	[0.670, 5.364]	0.012	2.275	[0.087, 4.462]	0.042
Infected or not						
No	Reference					
Yes	4.028	[1.598, 6.458]	0.001	3.561	[1.295, 5.826]	0.002
Duration of segregation	0.039	[−0.008, 0.087]	0.100	0.056	[0.012, 0.100]	0.012
Perceived stress	0.547	[0.448, 0.645]	<0.001	0.540	[0.448, 0.633]	<0.001
Self-efficacy	0.034	[−0.066, 0.134]	0.503	0.082	[−0.011, 0.175]	0.084
Perceived social support	−0.013	[−0.058, 0.032]	0.574	−0.008	[−0.050, 0.034]	0.715
*R* ^2^	0.568			0.579		
Adjusted *R*^2^	0.528			0.540		
*F*	14.290^***^			14.906^***^		

^***^p < 0.001.

*B*, Unstandardized coefficient; CI, Confidence interval.

Education level was transformed into two dummy variables with high school or below as the reference group (university or college vs. high school or below, postgraduate or above vs. high school or below).

### Mediating effects testing

**Figure 2:**
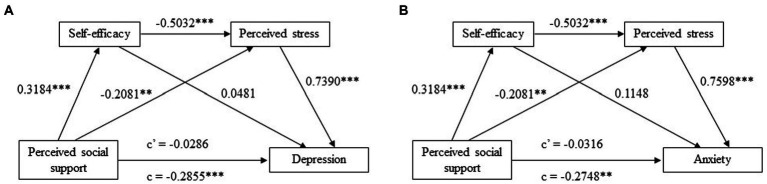
The chain mediation model with standardized path coefficients. ^**^, *p* < 0.01; ^***^, *p* < 0.001; c, total effect; c’, direct effect. **(A)** The chain mediating effect of self-efficacy and perceived stress between perceived social support and depression; **(B)** The chain mediating effect of self-efficacy and perceived stress between perceived social support and anxiety.

Taking into account the existence of significant correlations between depression, anxiety, perceived stress, self-efficacy and perceived social support, the nonparametric BC bootstrapping over 5,000 samples with 95% CI was employed to further test the chain mediating effect of self-efficacy and perceived stress on the association between perceived social support and depression (anxiety). The above demographic variables were treated as covariates in the mediation models. As indicated by Table 4; Figure 2, perceived stress played an intermediary role between perceived social support and depression (BC 95% CI [−0.1063, −0.0096]) with an effect size of −0.0547. While the mediating effect of self-efficacy between perceived social support and depression were not significant (BC 95% CI [−0.0111, 0.0224]), the chain mediating effect of self-efficacy and perceived stress between perceived social support and depression was estimated lie between −0.0717 and − 0.0163 with 95% confidence, which did not contain zero. It could be concluded that the chain mediating effect of self-efficacy and perceived stress between perceived social support and depression was significant with an estimated effect size of −0.0422. Combined with the total and direct effects, the results could be interpreted that individuals possessing higher level of perceived social support had higher self-efficacy and lower perceived stress, which in turn led to lower level of depression. Likewise, the mediating effect of perceived stress between perceived social support and anxiety was significant (BC 95% CI [−0.1034, −0.0091]) with an effect size of −0.0531. The chain mediating effect of self-efficacy and perceived stress between perceived social support and anxiety was significant (BC 95% CI [−0.0697, −0.0161]) with an effect size of −0.0409. Besides, the direct effect of perceived social support on depression and anxiety was not significant (BC 95% CI [−0.0545, 0.0341] and [−0.0519, 0.0307], respectively), indicating that self-efficacy and perceived stress completely mediated the relationship between perceived social support and depression (anxiety), and the proportion of indirect effect in total effect was 100%. Generally, the above findings confirmed that higher level of perceived social support generated higher self-efficacy and lower perceived stress, which alleviated the level of depression and anxiety among people isolated or quarantined under COVID-19 lockdown.

**Table 4:** Examination of chain mediating effects.

Model pathways	Effect	Boot SE	Boot LLCI	Boot ULCI
Perceived social support (X) → self-efficacy (M1) → perceived stress (M2) → depression (Y)				
Direct effect	−0.0102	0.0224	−0.0545	0.0341
Indirect effect (total)	−0.0915	0.0262	−0.1431	−0.0414
X → M1 → Y	0.0055	0.0083	−0.0111	0.0224
X → M2 → Y	−0.0547	0.0245	−0.1063	−0.0096
X → M1 → M2 → Y	−0.0422	0.0140	−0.0717	−0.0163
Total effect	−0.1016	0.0293	−0.1596	−0.0437
Perceived social support (X) → self-efficacy (M1) → perceived stress (M2) → anxiety (Y)				
Direct effect	−0.0106	0.0209	−0.0519	0.0307
Indirect effect (total)	−0.0817	0.0247	−0.1317	−0.0343
X→M1→Y	0.0123	0.0082	−0.0023	0.0302
X→M2→Y	−0.0531	0.0238	−0.1034	−0.0091
X→M1→M2→Y	−0.0409	0.0134	−0.0697	−0.0161
Total effect	−0.0923	0.0274	−0.1465	−0.0382

Boot SE, Standard error under bias-corrected percentile bootstrap method. Boot LLCI = 95% confidence interval lower; Boot ULCL = 95% confidence interval upper. Demographic variables were treated as covariates in the mediation models. Number of bootstrap samples for percentile bootstrap confidence intervals is 5000.

## Discussion

The present study was the first to address the chain mediating effect of self-efficacy and perceived stress on the association between perceived social support and depression (anxiety) among populations isolated or quarantined in Shanghai lockdown induced by the COVID-19 pandemic. The results revealed that 12 and 10.8% people under isolation or quarantine reported elevated level of depression and anxiety symptoms, respectively. Higher levels of depression and anxiety were related to higher education level, being healthcare workers (compared with COVID-19 patients), being infected with COVID-19 and higher perceived stress level. Longer duration of isolation or quarantine was also identified as a risk factor for anxiety. In addition, self-efficacy and perceived stress significantly mediated the association between perceived social support and depression (anxiety).

The prevalence of probable depression and anxiety was estimated to be 12 and 10.8% in this study, which was relatively lower than findings reported by previous studies, among which the prevalence of depression or anxiety during the COVID-19 pandemic ranged from 20 to 45% across diverse populations and geographic areas (13, 20, 24, 54, 55). Additionally, a recent cross-sectional study on population mental health under Shanghai lockdown reported a higher prevalence of depression (25.9%) and anxiety (19.9%) (56). China’s experience in containment, treatment and vaccines in response to the COVID-19 challenge and nationwide efforts to fight against the pandemic might buffer the psychological stress induced by the lockdown. Furthermore, it was reflected that the communication between infected patients and healthcare workers at the centralized site was excellent and they were encouraged to take moderate exercise (such as Tai Chi) during that period, which might create positive effects on their mental and physical health. Nevertheless, it is worth noting that the current prevalence is still higher than the lifetime prevalence of depressive disorders in adults Chinese, which was estimated to be 6.8% (57). Several studies noted that isolation and quarantine might arise detrimental psychological and physical effects as a result of restricted physical activity and social interaction, as well as changes in routine practices (58, 59). Accordingly, it makes sense to identify mental distress and implement appropriate psychological interventions in time as soon as public emergencies happen.

The findings revealed that isolated or quarantined populations with higher education level were more apt to develop depression or anxiety symptoms in comparison with those with a high school education or less, which was in consistent with prior findings that higher education level was significantly related to psychological distress like depression and stress (60, 61). It could be conceived that people possessing higher education are likely to bear more burden on work, family or academic tasks, which contributed to their vulnerability to the impact of COVID-19. Conversely, it was suggested by other relevant studies that less educated was connected with elevated levels of depression and anxiety resulted from the COVID-19 (62, 63). A former study even found no significant relationship between education level and mental health issues among nurses under the COVID-19 pandemic (37). Moreover, no association was discovered between other demographic characteristics and levels of depression and anxiety in this study, despite the fact that demographic characteristics such as age and gender have been linked to the psychological impacts of COVID-19 in numerous studies (13, 64). Such contradictions highlight the need for additional research in the relevant field.

It was found that longer duration of segregation was related to higher level of anxiety, which tied well with studies wherein quarantine length was associated with depression or anxiety (12, 13). Importantly, Lu et al. observed a dynamic pattern of anxiety and depression levels in quarantined populations, finding that anxiety and depression levels significantly increased at the initial stage of the quarantine, followed by a gradual decline, and went back up again as the quarantine progressed beyond 14 days (65). Long duration of segregation might add to the uncertainty in the pandemic containment and worry about their own health among isolated or quarantined populations. In light of the emotion fluctuations, further study with multiple evaluations on depression and anxiety symptoms is needed.

Results showed that healthcare workers reported more severe depression and anxiety symptoms. It could construe that healthcare workers were faced with more challenges in diverse aspects of work and life: the responsibilities of treating infected patients to prevent the spread of the virus; developing proper short-term programs and long-term plans; the discomfort caused by medical protective equipment; the fear of being infected or family member infected; balancing work and family and so on (66, 67). Numerous studies have now found that healthcare workers suffer from varying extent of psychological distress during public health emergencies like COVID-19 pandemic and Severe acute respiratory syndrome (SARS) (68–70). Thus, appropriate and practical psychological interventions should be provided to healthcare workers engaged in the management of COVID-19 patients.

Not surprisingly, individuals infected with COVID-19 exhibited higher levels of depression and anxiety, which was generally in accordance with previous studies. A substantial body of studies have reported acute and long-term health consequences in COVID-19 patients, including depression and anxiety disorders (71–73). Additionally, it has been well documented that major infectious diseases, such as SARS and Middle East respiratory syndrome (MERS) may affect the health of infected patients both physically and psychologically, even after the acute infection has subsided (74–78). As a result, psychological rehabilitation of COVID-19 patients is worth for equal concern.

In our study, the results showed that perceived social support and self-efficacy were negatively correlated to depression and anxiety level, while perceived stress was positively correlated to depression and anxiety level in isolated or quarantined people under lockdown. Moreover, self-efficacy and perceived stress played a completely intermediary role between perceived social support and depression (anxiety). That is to say, individuals who perceived low levels of social support tended to be accompanied by low self-efficacy and high perceived stress, which gave rise to subsequent development of depression and anxiety. Here, perceived social support and self-efficacy could be recognized as protective factors for depression and anxiety symptoms, while high perceived stress was a risk factor. The level of perceived stress under the COVID-19 pandemic merited special attention. A study examining the stress and psychological impact in SARS patients at the peak of the outbreak found that stress level not only increased but also correlated with negative psychological effects (79). Previous study also demonstrated that perceived stress level significantly mediated the relationship between negative life events and depression (80, 81). As important parts of personal cognitive resources, it is widely believed that perceived social support and self-efficacy could buffer the detrimental effects of stress on psychological conditions (82–85). Ma et al. conducted a nation-wide survey on the mental health of college students during the COVID-19 pandemic in China, illustrating that students with low perceived social support were more likely to have anxiety and depressive symptoms (86). A recently published study showed a significant correlation between self-efficacy and depression, anxiety and stress in the context of COVID-19 (87). From this standpoint, it is meaningful and constructive to promote one’s perceived social support and self-efficacy when developing suggestions or interventions for alleviating depressive and anxiety symptoms in isolated or quarantined populations.

### Limitations

Findings from this study presented potentially significant contributions to understanding the role of perceived stress, self-efficacy and perceived social support in the development of negative psychological consequences under COVID-19 lockdown. In spite of this, the findings of this study should be viewed in light of several limitations. The major limitation was the administration of self-report measures for depression and anxiety symptoms. Notably, a more persuasive standard for making psychiatric diagnoses must contain a structured clinical interview. Another limitation involved the nature of a cross-sectional study, which lacked baseline data on levels of depression, anxiety and perceived stress prior to the implementation of lockdown measures. Finally, the actual prevalence in this study was relatively lower than the estimated prevalence used in sample size calculation, which might limit the accuracy of the results. Future research should be conducted with more high-quality designs and comprehensive assessments in order to identify psychological disorders in populations affected by COVID-19 pandemic.

### Conclusion

The present findings confirmed high-risk populations and associated factors for higher level of depression and anxiety among populations under isolation or quarantine, including risk factors (high education level, being infected, longer duration of segregation and high level of perceived stress) and protective factors (self-efficacy and perceived social support). To sum up, managing elevated mental health burden under the COVID-19 pandemic cannot be overlooked, and authorities must strengthen their mental health service response. Recommended psychological strategies should take on board suggestions to promote personal mental resources and target interventions to support individuals who are disturbed by various mental distresses.

## Data availability statement

The raw data supporting the conclusions of this article will be made available by the authors, without undue reservation.

## Ethics statement

The studies involving human participants were reviewed and approved by the Ethics Committee of Naval Medical University. The patients/participants provided their written informed consent to participate in this study.

## Author contributions

LS, XW, YH, YL, and YW designed the study and wrote the protocols. YH, CL, WZ, PL, YX, and YC participated in the data collection and organization. LS and XW undertook the statistical analysis and wrote the manuscript, and then all authors participated in the revision. All authors are accountable for all aspects of the work in ensuring that questions related to the accuracy or integrity of any part of the work are appropriately investigated and resolved.

## Funding

This work was supported by the National Natural Science Foundation of China (no. 81771301) and the Military Project of China (nos. TJ2021018, PT202006).

## Conflict of interest

The authors declare that the research was conducted in the absence of any commercial or financial relationships that could be construed as a potential conflict of interest.

## Publisher’s note

All claims expressed in this article are solely those of the authors and do not necessarily represent those of their affiliated organizations, or those of the publisher, the editors and the reviewers. Any product that may be evaluated in this article, or claim that may be made by its manufacturer, is not guaranteed or endorsed by the publisher.

## Footnotes

^1^https://www.wjx.cn

## References

[1] World Health Organization. WHO Director-General's Opening Remarks at the Media Briefing on Covid-19 -11 march 2020 [online]. Geneva: World Health Organization (2020).

[2] Zhang X Zhang W Chen S. Shanghai's life-saving efforts against the current omicron wave of the Covid-19 pandemic. Lancet. (2022b) 399:2011–2. doi: 10.1016/S0140-6736(22)00838-8, PMID: 35533708 PMC9075855

[3] Chen Z Deng X Fang L Sun K Wu Y Che T. Epidemiological characteristics and transmission dynamics of the outbreak caused by the Sars-Cov-2 omicron variant in Shanghai, China: a descriptive study. Lancet Reg Health West Pac. (2022) 29:100592. doi: 10.1016/j.lanwpc.2022.100592, PMID: 36090701 PMC9448412

[4] World Health Organization. Infection prevention and control in the context of coronavirus disease (Covid-19): A living guideline, 7 march 2022. Geneva: World Health Organization (2022). 35767666

[5] Kraemer MUG Yang CH Gutierrez B Wu CH Klein B Pigott DM. The effect of human mobility and control measures on the Covid-19 epidemic in China. Science. (2020) 368:493–7. doi: 10.1126/science.abb4218, PMID: 32213647 PMC7146642

[6] Warren GW Lofstedt R Wardman JK. Covid-19: the winter lockdown strategy in five European nations. J Risk Res. (2021) 24:267–93. doi: 10.1080/13669877.2021.1891802

[7] Yang H Nie H Zhou D Wang Y Zuo W. The effect of strict lockdown on omicron Sars-Cov-2 variant transmission in Shanghai. Vaccines (Basel). (2022) 10:1392. doi: 10.3390/vaccines10091392, PMID: 36146469 PMC9500677

[8] Brooks SK Webster RK Smith LE Woodland L Wessely S Greenberg N. The psychological impact of quarantine and how to reduce it: rapid review of the evidence. Lancet. (2020) 395:912–20. doi: 10.1016/S0140-6736(20)30460-8, PMID: 32112714 PMC7158942

[9] Eyawo O Viens AM Ugoji UC. Lockdowns and low-and middle-income countries: building a feasible, effective, and ethical Covid-19 response strategy. Glob Health. (2021) 17:13. doi: 10.1186/s12992-021-00662-y, PMID: 33472638 PMC7816147

[10] Joffe AR. Covid-19: rethinking the lockdown groupthink. Front Public Health. (2021) 9:625778. doi: 10.3389/fpubh.2021.625778, PMID: 33718322 PMC7952324

[11] World Health Organization. Considerations for quarantine of individuals in the context of containment for coronavirus disease (Covid-19): Interim guidance, 19 march 2020. Geneva: World Health Organization (2020).

[12] Hou L Long F Meng Y Cheng X Zhang W Zhou R. The relationship between quarantine length and negative affect during the Covid-19 epidemic among the general population in China: the roles of negative cognition and protective factors. Front Psychol. (2021) 12:575684. doi: 10.3389/fpsyg.2021.575684, PMID: 33995168 PMC8113411

[13] Jassim G Jameel M Brennan E Yusuf M Hasan N Alwatani Y. Psychological impact of Covid-19, isolation, and quarantine: a cross-sectional study. Neuropsychiatr Dis Treat. (2021) 17:1413–21. doi: 10.2147/NDT.S311018, PMID: 34007180 PMC8123965

[14] Rossi R Socci V Talevi D Mensi S Niolu C Pacitti F. Covid-19 pandemic and lockdown measures impact on mental health among the general population in Italy. Front Psych. (2020) 11:790. doi: 10.3389/fpsyt.2020.00790, PMID: 32848952 PMC7426501

[15] Sun L Sun Z Wu L Zhu Z Zhang F Shang Z. Prevalence and risk factors for acute posttraumatic stress disorder during the Covid-19 outbreak. J Affect Disord. (2021) 283:123–9. doi: 10.1016/j.jad.2021.01.050, PMID: 33548905 PMC7840403

[16] Sun N Wei L Wang H Wang X Gao M Hu X. Qualitative study of the psychological experience of Covid-19 patients during hospitalization. J Affect Disord. (2021) 278:15–22. doi: 10.1016/j.jad.2020.08.040, PMID: 32949869 PMC7444461

[17] Santomauro DF Mantilla Herrera AM Shadid J Zheng P Ashbaugh C Pigott DM. Global prevalence and burden of depressive and anxiety disorders in 204 countries and territories in 2020 due to the Covid-19 pandemic. Lancet. (2021) 398:1700–12. doi: 10.1016/S0140-6736(21)02143-7, PMID: 34634250 PMC8500697

[18] Collaborators GMD. Global, regional, and National Burden of 12 mental disorders in 204 countries and territories, 1990–2019: a systematic analysis for the global burden of disease study 2019. Lancet Psychiatry. (2022) 9:137–50. doi: 10.1016/S2215-0366(21)00395-3 35026139 PMC8776563

[19] Khubchandani J Sharma S Webb FJ Wiblishauser MJ Bowman SL. Post-lockdown depression and anxiety in the Usa during the Covid-19 pandemic. J Public Health. (2021) 44:1–8. doi: 10.1093/pubmed/fdaa250 PMC7928742 33426559

[20] Dettmann LM Adams S Taylor G. Investigating the prevalence of anxiety and depression during the first Covid-19 lockdown in the United Kingdom: systematic review and meta-analyses. Br J Clin Psychol. (2022) 61:757–80. doi: 10.1111/bjc.12360, PMID: 35137427 PMC9111383

[21] Penninx B Benros ME Klein RS Vinkers CH. How Covid-19 shaped mental health: from infection to pandemic effects. Nat Med. (2022) 28:2027–37. doi: 10.1038/s41591-022-02028-2 36192553 PMC9711928

[22] Lazarus RS. Emotion and Adaption. Oxford: Oxford University Press (1991).

[23] Hu D Kong Y Li W Han Q Zhang X Zhu LX. Frontline Nurses' burnout, anxiety, depression, and fear statuses and their associated factors during the Covid-19 outbreak in Wuhan, China: a large-scale cross-sectional study. Eclinicalmedicine. (2020) 24:100424. doi: 10.1016/j.eclinm.2020.100424, PMID: 32766539 PMC7320259

[24] Liu CH Zhang E Wong GTF Hyun S Hahm HC. Factors associated with depression, anxiety, and Ptsd symptomatology during the Covid-19 pandemic: clinical implications for U.S. young adult mental health. Psychiatry Res. (2020) 290:113172. doi: 10.1016/j.psychres.2020.113172, PMID: 32512357 PMC7263263

[25] Roohafza HR Afshar H Keshteli AH Mohammadi N Feizi A Taslimi M. What's the role of perceived social support and coping styles in depression and anxiety? JRMS. (2014) 19:944–9. PMID: 25538777 PMC4274570

[26] House JS Umberson D Landis KR. Structures and processes of social support. Annu Rev Sociol. (1988) 14:293–318. doi: 10.1146/annurev.so.14.080188.001453

[27] Gariepy G Honkaniemi H Quesnel-Vallee A. Social support and protection from depression: systematic review of current findings in Western countries. Br J Psychiatry. (2016) 209:284–93. doi: 10.1192/bjp.bp.115.169094, PMID: 27445355

[28] Haber MG Cohen JL Lucas T Baltes BB. The relationship between self-reported received and perceived social support: a meta-analytic review. Am J Community Psychol. (2007) 39:133–44. doi: 10.1007/s10464-007-9100-9, PMID: 17308966

[29] Bandura A. Self-efficacy: toward a unifying theory of behavioral change. Psychol Rev. (1977) 84:191–215. doi: 10.1037/0033-295X.84.2.191, PMID: 847061

[30] Hubner T Wolfgang T Theis AC Steber M Wiedenmann L Wockel A. The impact of the Covid-19 pandemic on stress and other psychological factors in pregnant women giving birth during the first wave of the pandemic. Reprod Health. (2022) 19:189. doi: 10.1186/s12978-022-01493-9, PMID: 36064560 PMC9444078

[31] Simonetti V Durante A Ambrosca R Arcadi P Graziano G Pucciarelli G. Anxiety, sleep disorders and self-efficacy among nurses during Covid-19 pandemic: a large cross-sectional study. J Clin Nurs. (2021) 30:1360–71. doi: 10.1111/jocn.15685, PMID: 33534934 PMC8012992

[32] Wang L Luo J Li Y Zhou Y Wang W. Social support, anxiety, and depression in patients with prostate cancer: complete mediation of self-efficacy. Support Care Cancer. (2022) 30:6851–6. doi: 10.1007/s00520-022-07065-8, PMID: 35536329

[33] TMGH-Global COVID-19 Collaborative. Perceived stress of quarantine and isolation during Covid-19 pandemic: a global survey. Front Psych. (2021) 12:656664. doi: 10.3389/fpsyt.2021.656664 PMC8186534 34113270

[34] Nkire N Mrklas K Hrabok M Gusnowski A Vuong W Surood S. Covid-19 pandemic: demographic predictors of self-isolation or self-quarantine and impact of isolation and quarantine on perceived stress, anxiety, and depression. Front Psychiatry. (2021) 12:553468. doi: 10.3389/fpsyt.2021.553468, PMID: 33597900 PMC7882620

[35] Pereira-Morales AJ Adan A Forero DA. Perceived stress as a mediator of the relationship between neuroticism and depression and anxiety symptoms. Curr Psychol. (2017) 38:66–74. doi: 10.1007/s12144-017-9587-7

[36] Chang MW Brown R Wegener DT. Perceived stress linking psychosocial factors and depressive symptoms in low-income mothers. BMC Public Health. (2021) 21:62. doi: 10.1186/s12889-020-10118-4, PMID: 33407305 PMC7789186

[37] Pouralizadeh M Bostani Z Maroufizadeh S Ghanbari A Khoshbakht M Alavi SA. Anxiety and depression and the related factors in nurses of Guilan university of medical sciences hospitals during Covid-19: a web-based cross-sectional study. Int J Afr Nurs Sci. (2020) 13:100233. doi: 10.1016/j.ijans.2020.100233 32837911 PMC7417274

[38] Shah SMA Mohammad D Qureshi MFH Abbas MZ Aleem S. Prevalence, psychological responses and associated correlates of depression, anxiety and stress in a global population, during the coronavirus disease (Covid-19) pandemic. Community Ment Health J. (2021) 57:101–10. doi: 10.1007/s10597-020-00728-y, PMID: 33108569 PMC7590908

[39] Qiu J Shen B Zhao M Wang Z Xie B Xu Y. A Nationwide survey of psychological distress among Chinese people in the Covid-19 epidemic: implications and policy recommendations. General Psychiatry. (2020) 33:E100213. doi: 10.1136/gpsych-2020-100213, PMID: 32215365 PMC7061893

[40] Kroenke K Spitzer RL Williams JBW. The Phq-9: validity of a brief depression severity measure. J Gen Intern Med. (2001) 16:606–13. doi: 10.1046/j.1525-1497.2001.016009606.x, PMID: 11556941 PMC1495268

[41] Wang W Bian Q Zhao Y Li X Wang W Du J. Reliability and validity of the Chinese version of the patient health questionnaire (Phq-9) in the general population. Gen Hosp Psychiatry. (2014) 36:539–44. doi: 10.1016/j.genhosppsych.2014.05.021, PMID: 25023953

[42] Spitzer RL Kroenke K Williams JBW Löwe B. A brief measure for assessing generalized anxiety disorder: the gad-7. Arch Intern Med. (2006) 166:1092–7. doi: 10.1001/archinte.166.10.1092 16717171

[43] He XY Li CB Qian J Cui HS Wu WY. Reliability and validity of a generalized anxiety scale in general hospital outpatients. Shanghai Arch Psychiatry. (2010) 22:200–3.

[44] Cohen S Williamson G. Perceived stress in a probability sample of the United States In: Spacapan S Oskamp S, editors. The Social Psychology of Health. Newbury Park, Ca: Sage (1988)

[45] Lee EH. Review of the psychometric evidence of the perceived stress scale. Asian Nurs Res (Korean Soc Nurs Sci). (2012) 6:121–7. doi: 10.1016/j.anr.2012.08.004, PMID: 25031113

[46] Leung DY Lam T Chan SS. Three versions of perceived stress scale: validation in a sample of Chinese cardiac patients who smoke. BMC Public Health. (2010) 10:513. doi: 10.1186/1471-2458-10-513, PMID: 20735860 PMC2939644

[47] Schwarzer R Jerusalem M. Generalized self-efficacy scale In: Weinman JS Wright S Johnston M, editors. Measures in Health Psychology: A User’s portfolio. Causal and Control Beliefs. Windsor: Nfer-Nelson (1995)

[48] Zhang JX Schwarzer R. Measuring optimistic self-beliefs: a Chinese adaptation of the general self-efficacy scale. Psychologia. (1995) 38:174–81. doi: 10.3969/j.issn.1002-0829.2010.04.002

[49] Jiang QJ. Perceived social support scale. Chinese Behav Med Sci. (2001) 10:41–3.

[50] Zimet GD Dahlem NW Zimet SG Farley GK. The multidimensional scale of perceived social support. J Pers Assess. (1988) 52:30–41. doi: 10.1207/s15327752jpa5201_2 2280326

[51] Clara IP Cox BJ Enns MW Murray LT Torgrudc LJ. Confirmatory factor analysis of the multidimensional scale of perceived social support in clinically distressed and student samples. J Pers Assess. (2003) 81:265–70. doi: 10.1207/S15327752JPA8103_09, PMID: 14638451

[52] Preacher KJ Hayes AF. Spss and Sas procedures for estimating indirect effects in simple mediation models. Behav Res Methods Instrum Comput. (2004) 36:717–31. doi: 10.3758/BF03206553, PMID: 15641418

[53] Preacher KJ Hayes AF. Asymptotic and resampling strategies for assessing and comparing indirect effects in multiple mediator models. Behav Res Methods. (2008) 40:879–91. doi: 10.3758/BRM.40.3.879, PMID: 18697684

[54] Dragioti E Li H Tsitsas G Lee KH Choi J Kim J. A large-scale meta-analytic atlas of mental health problems prevalence during the Covid-19 early pandemic. J Med Virol. (2022) 94:1935–49. doi: 10.1002/jmv.27549, PMID: 34958144 PMC9015528

[55] Zhang SX Batra K Xu W Liu T Dong RK Yin A. Mental disorder symptoms during the Covid-19 pandemic in Latin America -a systematic review and meta-analysis. Epidemiol Psychiatr Sci. (2022a) 31:E23. doi: 10.1017/S2045796021000767, PMID: 35438066 PMC9069590

[56] Hall BJ Li G Chen W Shelley D Tang W. Prevalence of depression, anxiety, and suicidal ideation during the Shanghai 2022 lockdown: a cross-sectional study. J Affect Disord. (2023) 330:283–90. doi: 10.1016/j.jad.2023.02.121, PMID: 36863472 PMC9972774

[57] Lu J Xu X Huang Y Li T Ma C Xu G. Prevalence of depressive disorders and treatment in China: a cross-sectional epidemiological study. Lancet Psychiatry. (2021) 8:981–90. doi: 10.1016/S2215-0366(21)00251-0, PMID: 34559991

[58] Fuzeki E Groneberg DA Banzer W. Physical activity during Covid-19 induced lockdown: recommendations. J Occup Med Toxicol. (2020) 15:25. doi: 10.1186/s12995-020-00278-9, PMID: 32817753 PMC7422663

[59] Nie Y Ma Y Wu Y Li J Liu T Zhang C. Association between physical exercise and mental health during the Covid-19 outbreak in China: a Nationwide cross-sectional study. Front Psych. (2021) 12:722448. doi: 10.3389/fpsyt.2021.722448, PMID: 34489763 PMC8418057

[60] Demilew D Angaw DA Getnet B Tesfaye B Atnafu A Andualem Z. Psychological distress and associated factors among healthcare professionals in Ethiopia during the Covid-19 pandemic: a cross-sectional study. BMJ Open. (2022) 12:E057197. doi: 10.1136/bmjopen-2021-057197, PMID: 35902189 PMC9340579

[61] Johnson-Lawrence V Scott JB James SA. Education, perceived discrimination and risk for depression in a southern black cohort. Aging Ment Health. (2020) 24:1872–8. doi: 10.1080/13607863.2019.1647131, PMID: 31389255 PMC7004854

[62] Coley RL Baum CF. Trends in mental health symptoms, service use, and unmet need for services among U.S. adults through the first 9 months of the Covid-19 pandemic. Transl Behav Med. (2021) 11:1947–56. doi: 10.1093/tbm/ibab030, PMID: 33823047 PMC8139151

[63] Coley RL Carey N Baum CF Hawkins SS. Covid-19-related stressors and mental health disorders among us adults. Public Health Rep. (2022) 137:1217–26. doi: 10.1177/00333549221120451, PMID: 36073255 PMC9459370

[64] Gabarrell-Pascuet A Koyanagi A Félez-Nobrega M Cristóbal-Narváez P Mortier P Vilagut G. The association of age with depression, anxiety, and posttraumatic stress symptoms during the Covid-19 pandemic in Spain: the role of loneliness and pre-pandemic mental disorder. Psychosom Med. (2022) 85:42–52. doi: 10.1097/PSY.0000000000001146 36201774

[65] Lu W. Yuan L. Xu J. Xue F. Zhao B. Webster C. (2020). The psychological effects of quarantine during Covid-19 outbreak: Sentiment analysis of social media data. Available at: Https://Ssrn.Com/Abstract=3627268

[66] Shreffler J Petrey J Huecker M. The impact of Covid-19 on healthcare worker wellness: a scoping review. West J Emerg Med. (2020) 21:1059–66. doi: 10.5811/westjem.2020.7.48684, PMID: 32970555 PMC7514392

[67] Wang H Liu Y Hu K Zhang M Du M Huang H. Healthcare Workers' stress when caring for Covid-19 patients: an altruistic perspective. Nurs Ethics. (2020) 27:1490–500. doi: 10.1177/0969733020934146, PMID: 32662326

[68] Gonzalez Mendez MJ Ma L Alvarado R Ramirez J Xu KP Xu HF. A multi-center study on the negative psychological impact and associated factors in Chinese healthcare workers 1 year after the Covid-19 initial outbreak. Int J Public Health. (2022) 67:1604979. doi: 10.3389/ijph.2022.1604979, PMID: 36090824 PMC9454095

[69] Liu S Han W Shen C Zhu C Wang Q Liang X. Depressive state in the emergency department during Covid-19: a National Cross-Sectional Survey in China. Front Psych. (2021) 12:566990. doi: 10.3389/fpsyt.2021.566990, PMID: 34194341 PMC8236535

[70] Wu P Fang Y Guan Z Fan B Kong J Yao Z. The psychological impact of the Sars epidemic on hospital employees in China: exposure, risk perception, and altruistic acceptance of risk. Can J Psychiatry. (2009) 54:302–11. doi: 10.1177/070674370905400504, PMID: 19497162 PMC3780353

[71] Huang C Huang L Wang Y Li X Ren L Gu X. 6-month consequences of Covid-19 in patients discharged from hospital: a cohort study. Lancet. (2021) 397:220–32. doi: 10.1016/S0140-6736(20)32656-8, PMID: 33428867 PMC7833295

[72] Huang L Yao Q Gu X Wang Q Ren L Wang Y. 1-year outcomes in hospital survivors with Covid-19: a longitudinal cohort study. Lancet. (2021) 398:747–58. doi: 10.1016/S0140-6736(21)01755-4, PMID: 34454673 PMC8389999

[73] Magnúsdóttir I Lovik A Unnarsdóttir AB Mccartney D Ask H Kõiv K. Acute Covid-19 severity and mental health morbidity trajectories in patient populations of six nations: an observational study. Lancet Public Health. (2022) 7:E406–16. doi: 10.1016/S2468-2667(22)00042-1, PMID: 35298894 PMC8920517

[74] Almekhlafi GA Albarrak MM Mandourah Y Hassan S Alwan A Abudayah A. Presentation and outcome of Middle East respiratory syndrome in Saudi intensive care unit patients. Crit Care. (2016) 20:123. doi: 10.1186/s13054-016-1303-8, PMID: 27153800 PMC4859954

[75] Chan K Zheng J Mok Y Li Y Liu Y Chu C. Sars: Prognosis, Outcome And Sequelae. Respirology. (2010) 8:S36–40. doi: 10.1046/j.1440-1843.2003.00522.x PMC7169213 15018132

[76] Cheng SK Wong CW Tsang J Wong KC. Psychological distress and negative appraisals in survivors of severe acute respiratory syndrome (Sars). Psychol Med. (2004) 34:1187–95. doi: 10.1017/S0033291704002272, PMID: 15697045

[77] Lee SH Shin HS Park HY Kim JL Lee JJ Lee H. Depression as a mediator of chronic fatigue and post-traumatic stress symptoms in Middle East respiratory syndrome survivors. Psychiatry Investig. (2019) 16:59–64. doi: 10.30773/pi.2018.10.22.3, PMID: 30605995 PMC6354037

[78] Moldofsky H Patcai J. Chronic widespread musculoskeletal pain, fatigue, depression and disordered sleep in chronic post-Sars syndrome; a case-controlled study. BMC Neurol. (2011):11. doi: 10.1186/1471-2377-11-37 21435231 PMC3071317

[79] Chua SE Cheung V McAlonan GM Cheung C Wong JWS Cheung EPT. Stress and psychological impact on Sars patients during the outbreak. Can J Psychiatr. (2004) 49:385–90. doi: 10.1177/070674370404900607 15283533

[80] Ioannou M Kassianos AP Symeou M. Coping with depressive symptoms in young adults: perceived social support protects against depressive symptoms only under moderate levels of stress. Front Psychol. (2018) 9:2780. doi: 10.3389/fpsyg.2018.02780 30692958 PMC6340372

[81] Kuiper NA Olinger LJ Lyons LM. Global perceived stress level as a moderator of the relationship between negative life events and depression. J Hum Stress. (1986) 12:149–53. doi: 10.1080/0097840X.1986.9936781, PMID: 3559198

[82] Reid KM Taylor MG. Social support, stress, and maternal postpartum depression: a comparison of supportive relationships. Soc Sci Res. (2015) 54:246–62. doi: 10.1016/j.ssresearch.2015.08.009, PMID: 26463547

[83] Santini ZI Koyanagi A Tyrovolas S Mason C Haro JM. The association between social relationships and depression: a systematic review. J Affect Disord. (2015) 175:53–65. doi: 10.1016/j.jad.2014.12.049 25594512

[84] Shao R He P Ling B Tan L Xu L Hou Y. Prevalence of depression and anxiety and correlations between depression, anxiety, family functioning, social support and coping styles among Chinese medical students. Bmc Psychol. (2020) 8:38. doi: 10.1186/s40359-020-00402-8, PMID: 32321593 PMC7178943

[85] Wang J Mann F Lloyd-Evans B Ma R Johnson S. Associations between loneliness and perceived social support and outcomes of mental health problems: a systematic review. BMC Psychiatry. (2018) 18:156. doi: 10.1186/s12888-018-1736-5, PMID: 29843662 PMC5975705

[86] Ma Z Zhao J Li Y Chen D Wang T Zhang Z. Mental health problems and correlates among 746 217 college students during the coronavirus disease 2019 outbreak in China. Epidemiol Psychiatr Sci. (2020) 29:E181. doi: 10.1017/S2045796020000931, PMID: 33185174 PMC7681173

[87] Haywood D Mason O. Perception of Covid-19 threat, low self-efficacy, and external locus of control Lead to psychological distress during the Covid-19 pandemic. Psychol Health Med. (2022) 15:1–8. doi: 10.1080/13548506.2022.2124290 36111351

