# Supplementary material for: COVID-19 pandemic-related depression and anxiety under lockdown: The chain mediating effect of self-efficacy and perceived stress
Source: Front Psychiatry. 2023 Apr 26;14:1100242. doi: 10.3389/fpsyt.2023.1100242 (PMC10169693; doi:10.3389/fpsyt.2023.1100242)
Supplement: Supplementary file 1 [file Table_1.docx]

| **Table S1 Influence of demographic characteristics on depression and anxiety level** | | | | | | | | |
| --- | --- | --- | --- | --- | --- | --- | --- | --- |
|  |  | Depression level | | |  | Anxiety level | | |
|  |  | PHQ-9 score M (SD) | F/*t*/Z | *p* value |  | GAD-7 score M (SD) | F/*t*/Z | *p* value |
| Age (years) ^a^ |  |  | 0.714 | 0.491 |  |  | 0.016 | 0.985 |
| 18-44 |  | 5.36 (4.71) |  |  |  | 4.16 (4.37) |  |  |
| 45-59 |  | 4.56 (4.82) |  |  |  | 4.16 (4.84) |  |  |
| 60-74 |  | 3.78 (4.24) |  |  |  | 3.89 (4.57) |  |  |
| Gender ^b^ |  |  | 0.720 | 0.473 |  |  | -1.303 | 0.194 |
| Male |  | 5.49 (4.34) |  |  |  | 3.57 (4.20) |  |  |
| Female |  | 4.95 (4.90) |  |  |  | 4.49 (4.54) |  |  |
| Education level ^a^ |  |  | 0.975 | 0.379 |  |  | 2.592 | 0.078 |
| High school or below | | 4.45 (3.80) |  |  |  | 3.02 (3.54) |  |  |
| University or college | | 5.66 (5.43) |  |  |  | 4.28 (4.86) |  |  |
| Postgraduate or above | | 5.07 (4.20) |  |  |  | 5.07 (4.35) |  |  |
| Marital status ^b^ |  |  | 1.813 | 0.072 |  |  | 0.821 | 0.413 |
| Unmarried |  | 5.84 (4.93) |  |  |  | 4.44 (4.60) |  |  |
| Married | | 4.53 (4.40) |  |  |  | 3.87 (4.28) |  |  |
| Employment status ^c^ |  |  | -0.276 | 0.783 |  |  | -0.038 | 0.969 |
| Employed |  | 5.21 (4.72) |  |  |  | 4.15 (4.49) |  |  |
| Unemployed |  | 4.94 (4.67) |  |  |  | 4.12 (4.26) |  |  |
| Smoking status ^c^ |  |  | -0.548 | 0.584 |  |  | -1.092 | 0.275 |
| Nonsmoker |  | 5.06 (4.63) |  |  |  | 4.28 (4.49) |  |  |
| Smoker |  | 5.69 (5.07) |  |  |  | 3.38 (4.09) |  |  |
| Presence of family or pets ^b^ | | | -0.580 | 0.562 |  |  | -0.584 | 0.560 |
| Yes |  | 4.92 (5.29) |  |  |  | 3.92 (4.40) |  |  |
| No |  | 5.34 (4.18) |  |  |  | 4.32 (4.47) |  |  |
| Current status ^b^ |  |  | -0.510 | 0.611 |  |  | -2.191 | 0.030 |
| Patients |  | 4.95 (4.64) |  |  |  | 3.30 (4.28) |  |  |
| Healthcare workers |  | 5.32 (4.75) |  |  |  | 4.80 (4.45) |  |  |
| Infected or not ^c^ |  |  | -0.003 | 0.997 |  |  | -2.130 | 0.033 |
| No |  | 5.08 (4.48) |  |  |  | 4.62 (4.35) |  |  |
| Yes |  | 5.25 (4.96) |  |  |  | 3.58 (4.48) |  |  |
| Duration of segregation (days) ^b^ | | | -1.605 | 0.110 |  |  | -3.092 | 0.002 |
| ≤ 14 |  | 4.46 (4.28) |  |  |  | 2.90 (3.53) |  |  |
| > 14 |  | 5.64 (4.92) |  |  |  | 5.00 (4.78) |  |  |
| Note: ^a^, one-way analysis of variance; ^b^, *t*-test; ^c^, Mann–Whitney *U* test. | | | | | | | | |
| M = Mean; SD = Standard deviation; PHQ-9 = Patient Health Questionnaire-9; GAD-7 = Generalized Anxiety Disorder-7 | | | | | | | | |
